# Supplementary material for: Analysis of Genetic Diversity and Population Structure of Endemic Endangered Goose (Anser cygnoides) Breeds Based on Mitochondrial CYTB
Source: Animals (Basel). 2024 May 16;14(10):1480. doi: 10.3390/ani14101480 (PMC11117250; doi:10.3390/ani14101480)
Supplement: Supplementary file 1 [file animals-14-01480-s001.zip › Supplementary materials -Table S1.pdf]

Table S1. Detailed information on six endangered goose breeds.

LX

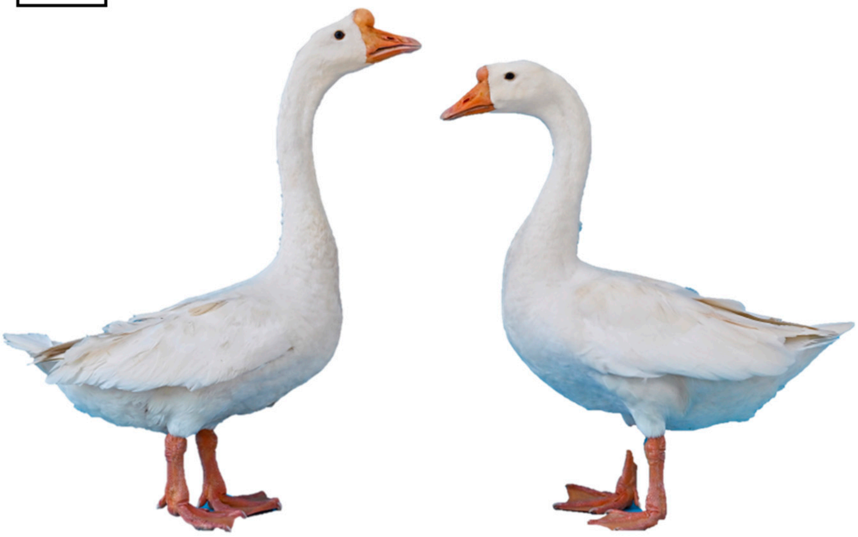

Breed: Lingxiang White goose (**LX**)
Birthplace: Zhuzhou County (Hunan, China)
Geographical position: E 112°57′, N 26°03′
Appearance characteristics: Lingxiang white geese are small in size. The whole-body feathers are white, and some geese have a cluster of crown hair on their heads. The beak is orange in color and the tip of the beak is lighter. The rainbow color is blue gray, the skin is light yellow, and the shins and fins are orange. The male goose has obvious sarcoma on its head, which is round, smooth, and wrinkle free. The head of the female goose has a flat sarcoma, and the hindquarters are well developed.

YE

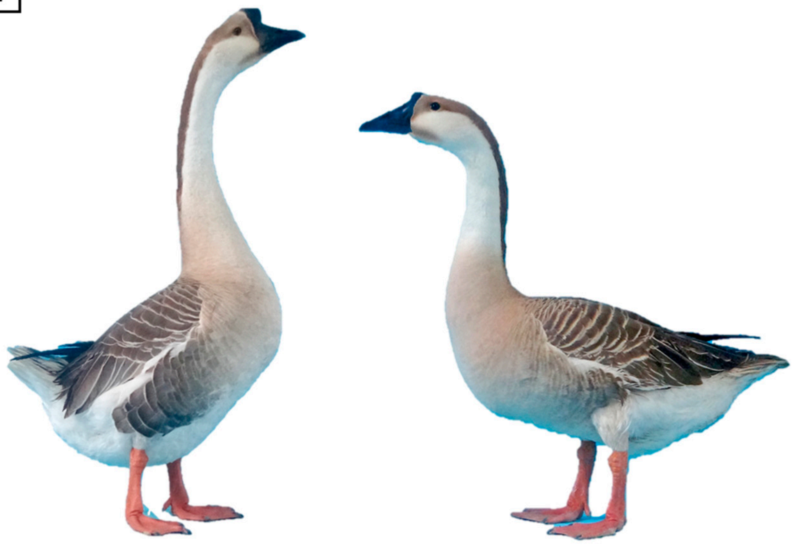

Breed: Yan goose (**YE**)
Birthplace: Lu'an County (Anhui, China)
Geographical position: E 115°22′, N 31°48′

Appearance characteristics: The body shape of Yan geese is relatively large and rectangular. Moderate in size, with a sarcoma on the head, a black beak, and orange red legs and fins. The whole body is covered with fur and wing feathers with brown gray edges, and the tail is white with gray black edges. The back of the head and neck starts from the bottom of the beak and eye corners, along the back of the neck until the base of the neck, there is a black mane like band that narrows from wide to narrow. The other feathers on the neck are gray white, the chest is gray brown, and the abdomen is white. Named after its fur resembling a wild goose.

YJ

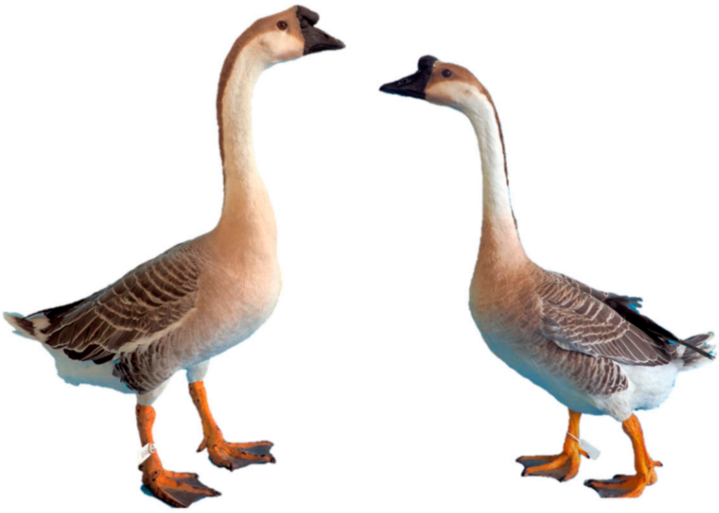

Breed: Yangjiang goose (**YJ**)
Birthplace: Yangjiang County (Guangdong, China)
Geographical position: E 111°16′, N 21°28′
Appearance characteristics: The Yangjiang goose has a delicate and compact body shape. There is a brownish yellow feather band from the top of the head to the back of the neck, resembling a horse's mane. The whole-body feathers are tightly attached, and the back, wings, and tail are brownish gray. The beak and sarcoma are black, while the tibia and fins are orange yellow. The mother goose has a slender head and a long neck, with a slightly tiled trunk and a gentle temperament; The male goose has a large head and a thick collar, with a slightly boat shaped trunk, and is clearly male. On the chest, back, wing-tail, and outer sides of the two calves, there is gray fur with white silver border feathers that are 0.1 centimeters wide at the edges of the fur.

WZ

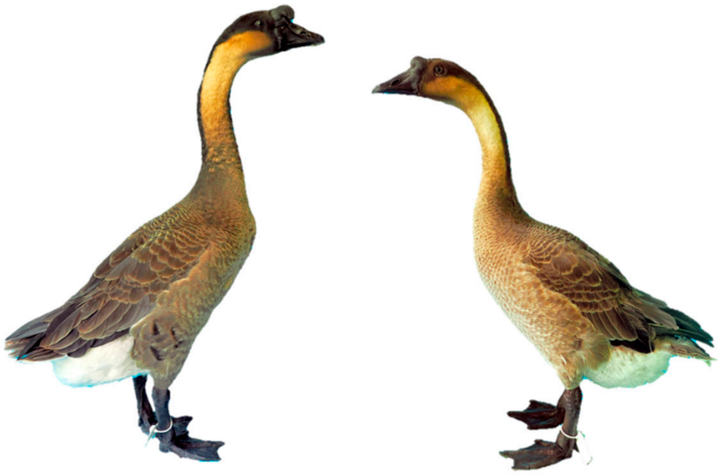

Breed: Wuzong goose (**WZ**)
Birthplace: Qingyuan County (Guangdong, China)
Geographical position: E 113°55′, N 23°26′
Appearance characteristics: Wuzong goose is a small breed of meat goose. Its body is compact, with a small head, a thin neck, and short legs; The male goose has a larger body size and is of the olive nucleus type; The mother goose is wedge-shaped; Most of the feathers are dark brown, with a mane like black brown feather band from the top of the head to the neck; The feathers on both sides of the neck are white, while the wing feathers, shoulder feathers, back feathers, and tail feathers are black. The ends of the feathers have obvious brown silver edges; There is a wide white feather band on both sides of the back, starting from the shoulder and ending at the base of the tail. Named after the fact that most of the feathers are dark brown.

XP

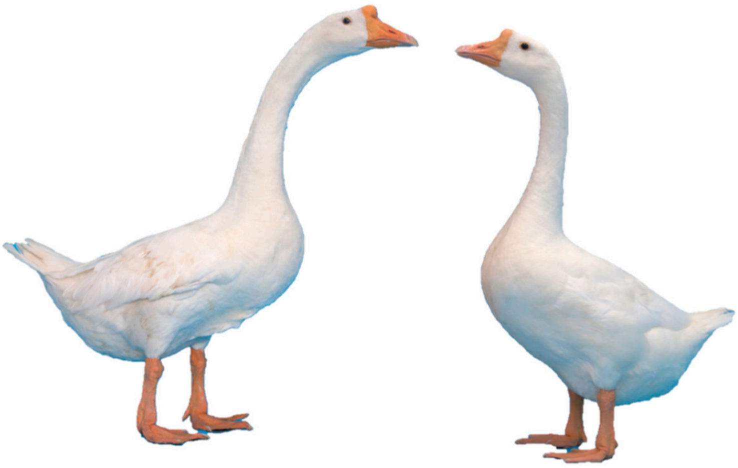

Breed: Xupu goose (**XP**)
Birthplace: Xupu County (Hunan, China)
Geographical position: E 110°15′, N 28°17′

Appearance characteristics: The Xupu goose is tall and has a slightly longer, cylindrical body. The male goose has a high head and neck, is upright and majestic, and its call is clear and loud, with strong herd protection. The female goose is slightly smaller in size, gentle in nature, and has strong foraging ability. During egg laying, the hindquarters are plump and round in shape. There are two main colors of feathers: white and gray, with white being the majority. The beak, sarcoma, tibia, and fins are all orange in color. The skin is light yellow, the eyelids are yellow, and the rainbow is grayish blue. The feathers of Xupu geese, as superior raw materials, have become a popular badminton production material for the Chinese national badminton team.

BZ

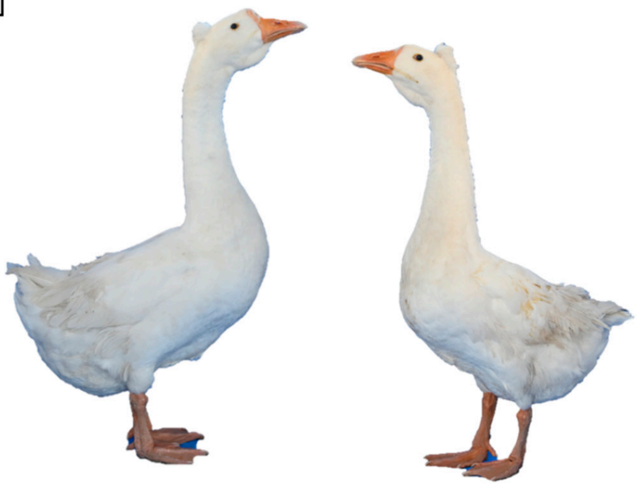

Breed: Baizi goose (**BZ**)
Birthplace: Jinxiang County (Shandong, China)
Geographical position: E 116°18′, N 35°04′
Appearance characteristics: The head size of the Baizi goose is moderate and delicate, with orange flesh tumors on the top of the head. The male goose has larger flesh tumors than the female goose, and there is no flesh drooping under the collar. The neck is thin and long, the chest is wide and protruding, the back is wide and flat, and the male goose's breasts are contracted. The mother goose has a drooping abdomen, a slightly elliptical body, a flat beak that is orange red, and fins that are orange red. Powerful in walking, with a thumping sound as it rushes, the head and neck are immediately raised, the posture is majestic, and the feathers of the whole body are pure white and shiny. Named after its annual production of 100 eggs.
