# Supplementary material for: Analysis of Genetic Diversity and Population Structure of Endemic Endangered Goose (Anser cygnoides) Breeds Based on Mitochondrial CYTB
Source: Animals (Basel). 2024 May 16;14(10):1480. doi: 10.3390/ani14101480 (PMC11117250; doi:10.3390/ani14101480)
Supplement: Supplementary file 1 [file animals-14-01480-s001.zip › Supplementary materials -Table S2.pdf]

Table S2. Distribution data of haplotypes of six endangered goose breeds:

Hap_1: 4 [YJ30-Yanjian YJ24-Yanjian YJ18-Yanjian WZ23-wuzong]

Hap_2: 1 [YJ29-Yanjian]

Hap_3: 5 [YJ28-Yanjian YJ23-Yanjian YJ15-Yanjian YJ7-Yanjian WZ30-wuzong]

Hap_4: 12 [YJ27-Yanjian YJ21-Yanjian YJ20-Yanjian YJ19-Yanjian YE28-Yan YE26-Yan YE20-Yan XP17-xupu WZ28-wuzong WZ27-wuzong LX28-linxiang LX20-linxiang]

Hap_5: 1 [YJ26-Yanjian]

Hap_6: 4 [YJ25-Yanjian YJ22-Yanjian XP30-xupu XP27-xupu]

Hap_7: 1 [YJ17-Yanjian]

Hap_8: 1 [YJ16-Yanjian]

Hap_9: 7 [YJ13-Yanjian YJ10-Yanjian YJ3-Yanjian YE7-Yan YE1-Yan BZ15-baizi BZ14-baizi]

Hap_10: 16 [YJ12-Yanjian YJ11-Yanjian YJ6-Yanjian YE14-Yan XP29-xupu XP7-xupu WZ12-wuzong WZ9-wuzong WZ8-wuzong WZ4-wuzong WZ2-wuzong LX15-linxiang LX10-linxiang BZ11-baizi BZ10-baizi BZ7-baizi]

Hap_11: 2 [YJ9-Yanjian LX4-linxiang]

Hap_12: 2 [YJ8-Yanjian YJ5-Yanjian]

Hap_13: 9 [YJ4-Yanjian YE15-Yan WZ20-wuzong LX30-linxiang BZ27-baizi BZ19-baizi BZ12-baizi BZ9-baizi BZ4-baizi]

Hap_14: 9 [YJ2-Yanjian YE8-Yan XP2-xupu WZ11-wuzong WZ6-wuzong WZ3-wuzong WZ1-wuzong LX8-linxiang LX2-linxiang]

Hap_15: 1 [YJ1-Yanjian]

Hap_16: 5 [YE30-Yan XP13-xupu XP5-xupu LX16-linxiang BZ23-baizi]

Hap_17: 1 [YE29-Yan]

Hap_18: 1 [YE27-Yan]

Hap_19: 3 [YE25-Yan XP24-xupu BZ24-baizi]

Hap_20: 1 [YE24-Yan]

Hap_21: 1 [YE23-Yan]

Hap_22: 1 [YE22-Yan]

Hap_23: 2 [YE21-Yan LX17-linxiang]

Hap_24: 1 [YE19-Yan]

Hap_25: 3 [YE18-Yan XP25-xupu XP23-xupu]

Hap_26: 2 [YE17-Yan LX21-linxiang]

Hap_27: 1 [YE16-Yan]

Hap_28: 3 [YE13-Yan LX12-linxiang BZ26-baizi]

Hap_29: 3 [YE12-Yan LX14-linxiang LX13-linxiang]

Hap_30: 3 [YE11-Yan XP12-xupu XP8-xupu]

Hap_31: 1 [YE10-Yan]

Hap_32: 1 [YE9-Yan]

Hap_33: 3 [YE6-Yan YE2-Yan XP4-xupu]

Hap_34: 2 [YE5-Yan XP3-xupu]

Hap_35: 2 [YE4-Yan LX6-linxiang]

Hap_36: 1 [YE3-Yan]

Hap_37: 1 [XP28-xupu]

Hap_38: 1 [XP26-xupu]
Hap_39: 1 [XP22-xupu]
Hap_40: 2 [XP21-xupu XP20-xupu]
Hap_41: 1 [XP19-xupu]
Hap_42: 1 [XP18-xupu]
Hap_43: 1 [XP15-xupu]
Hap_44: 1 [XP14-xupu]
Hap_45: 1 [XP11-xupu]
Hap_46: 3 [XP10-xupu WZ13-wuzong BZ18-baizi]
Hap_47: 1 [XP9-xupu]
Hap_48: 5 [XP6-xupu BZ8-baizi BZ3-baizi BZ2-baizi BZ1-baizi]
Hap_49: 1 [XP1-xupu]
Hap_50: 1 [WZ29-wuzong]
Hap_51: 2 [WZ26-wuzong WZ19-wuzong]
Hap_52: 1 [WZ25-wuzong]
Hap_53: 1 [WZ24-wuzong]
Hap_54: 1 [WZ22-wuzong]
Hap_55: 1 [WZ21-wuzong]
Hap_56: 1 [WZ18-wuzong]
Hap_57: 1 [WZ17-wuzong]
Hap_58: 1 [WZ16-wuzong]
Hap_59: 1 [WZ14-wuzong]
Hap_60: 4 [WZ10-wuzong LX11-linxiang BZ22-baizi BZ13-baizi]
Hap_61: 1 [WZ7-wuzong]
Hap_62: 1 [WZ5-wuzong]
Hap_63: 1 [LX29-linxiang]
Hap_64: 1 [LX27-linxiang]
Hap_65: 2 [LX26-linxiang LX25-linxiang]
Hap_66: 1 [LX24-linxiang]
Hap_67: 1 [LX23-linxiang]
Hap_68: 1 [LX22-linxiang]
Hap_69: 1 [LX19-linxiang]
Hap_70: 1 [LX18-linxiang]
Hap_71: 1 [LX9-linxiang]
Hap_72: 3 [LX7-linxiang LX5-linxiang BZ6-baizi]
Hap_73: 3 [LX3-linxiang LX1-linxiang BZ30-baizi]
Hap_74: 1 [BZ29-baizi]
Hap_75: 1 [BZ28-baizi]
Hap_76: 1 [BZ25-baizi]
Hap_77: 1 [BZ21-baizi]
Hap_78: 1 [BZ20-baizi]
Hap_79: 1 [BZ17-baizi]
Hap_80: 1 [BZ16-baizi]
Hap_81: 1 [BZ5-baizi]
